# Challenges in Diagnosis and Treatment of a Cervical Carcinoma Complicated by Genital Prolapse

**DOI:** 10.1155/2021/5523016

**Published:** 2021-08-21

**Authors:** Aikaterini-Eirini Evangelopoulou, Konstantinos Zacharis, Konstantina Balafa, Alexandros Daponte, Ourania Koukoura

**Affiliations:** Obstetrics and Gynecology Department, University Hospital of Larissa, Thessaly, Greece

## Abstract

Cervical squamous cell carcinoma of the uterus associated with pelvic organ prolapse is very rare and usually occurs in elderly women. We hereby present an 81-year-old postmenopausal woman presented to the outpatient department with an ulcerated irreducible uterine procidentia. The prolapse was reduced under general anesthesia and biopsy of the lesions confirmed a cervical squamous cell carcinoma. Pretreatment clinical staging revealed a 16 cm enlarged uterus and mild to moderate unilateral hydroureteronephrosis, secondary to periureteric infiltration, clinical stage IIIB. The patient was disqualified from surgery, and palliative chemotherapy plus radiotherapy was recommended. Patient's general condition was rapidly deteriorated, and three months after the diagnosis, the patient passed away. Therapeutic management of cervical cancer associated with uterovaginal prolapse is not well established. Hence, this article presents the clinical concerns that arise in such rare and neglected cases.

## 1. Introduction

Cervical squamous cell carcinoma and pelvic organ prolapse (POP) are individually considered common events in women of advanced age. However, the concurrence of complete uterine procidentia and cervical cancer is scarcely encountered, mainly in elderly women from low-income countries [1]. The exact incidence of uterine cervical cancer associated with uterine prolapse is unknown. The reported prevalence (0.14-1%) is primarily based on sporadic case reports [2–5]. In most of these cases, however, the pathophysiologic association of these entities was discussed, mainly focusing on modes of treatment. We herein present a unique case of a woman with a history of uterine prolapse who developed a huge malignant cervical tumor which led to an irreducible genital prolapse. The clinical dilemmas faced, and the management plan employed, are discussed in correlation with only a few similar cases already reported.

## 2. Case Presentation

An 81-year-old Caucasian woman gravida 2, para 2 presented to our outpatient department with new-onset vaginal bleeding and gradually worsening uterine prolapse for the past three months. She had a documented history of uterine prolapse for the past 15 years. The patient also described difficulty in passing urine for the last 5 years and worsening local pain. Her renal function was affected, and a urine catheter was inserted. She also reported well-controlled arterial hypertension and asthma.

**Figure 1:**
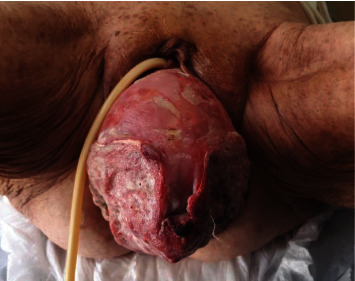
Cervical cancer in association with irreducible uterine prolapse.

The physical examination revealed uterine procidentia with an ulcerated lesion located on the posterior wall of the ectocervix (Figure 1). *Τ*he prolapsed uterus was irreducible, and attempts to manually reduce the prolapse were unsuccessful. Informed consent was obtained, and patient was transferred to theater for manual reduction of the prolapsed uterus under general anesthesia. The cervix was biopsied prior to the procedure.

Initial attempts failed to restore the uterus to its original position. Applying controlled pressure to the bulky part of the prolapsed uterus along with careful pushing of the cervix facilitate the replacement of the uterus into the vagina. A large vaginal swab was used to refrain the uterus from displacement. The histopathology report revealed a squamous cell carcinoma of the cervix.

**Figure 2:**
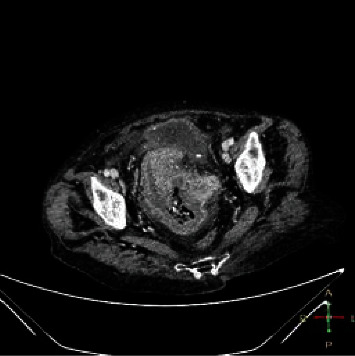
Axial pelvic computed tomography of the patient showing the enlarged uterus after reduction of the prolapse.

Pretreatment clinical staging tests including chest X-ray, ultrasound, and contrast-enhanced computed tomography scan of the abdomen were performed. Significant findings were a 16 cm enlarged uterus and mild to moderate unilateral hydroureteronephrosis, secondary to periureteric infiltration, clinical stage IIIB (Figure 2). Her chest X-ray did not show any abnormal features. Cystoscopy and proctoscopy were also performed for staging with no evidence of bladder or rectal invasion.

The patient was disqualified from surgery due to the severity of the disease (FIGO Stage IIIB). Palliative chemotherapy with cisplatin plus radiotherapy was recommended, but the patient's general condition was rapidly deteriorated, manifested by cachexia, anorexia, and pelvic pain; thus, treatment was postponed. Three months after the diagnosis, a telephone follow-up took place. We were informed that end-life care measures, regarding her comfort, were performed by an in-home nurse.

## 3. Discussion

POP incidence is estimated among 2.9-41.1%. The incidence of cervical cancer in women undergoing a vaginal hysterectomy for pelvic organ prolapse is 0.3% with no prior established diagnosis [4]. However, many studies do not distinguish between prolapse of all pelvic organs and prolapse of the uterus alone, which makes it difficult to determine the true incidence of the disease. POP can affect women of all ages but typically occurs in the elderly [5].

POP can cause symptoms such as sensation of pelvic pressure, bladder and bowel dysfunction, bulging through the introitus, and dyspareunia [6]. However, only 3% of patients report symptoms and many cases are not clinically appreciable during vaginal examination alone [7]. A common complication of procidentia is the ulceration of the most dependent area of the prolapse. A biopsy is warranted in any ulcerated lesion in order to exclude malignancy. Although our patient reported new-onset vaginal bleeding, the size of the malignant lesion indicated that the 81-year-old woman had probably overlook her symptoms (including bleeding) for a long period of time.

Although cervical cancer and prolapse are common diseases, the coincidence of both of them is rare [8] and occurs in women between 60 and 80 years old where the procidentia exists for 10 years or more [9]. On the other hand, cervical carcinoma peaks in the 5th decade of women's life [7, 10]. The different age distributions can explain, in part, the rarity of concurrence in which both entities coexist. Our patient reported the presence of uterine prolapse for over 15 years with new-onset vaginal bleeding. There are only a few cases in the literature describing cervical cancer in women with POP; therefore, there is no strong evidence that the recurrent injury of the cervical epithelium may cause neoplasm in the abovementioned patients [11, 12]. Whether screening for cervical cancer should be prolonged in patients with uterine procidentia is a question yet to be answered.

Management of patients with POP complicated with cervical cancer should be multidisciplinary due to concomitant diseases and involve medical subspecialists such as gynecologic oncologist, urogynecologist, and radiation oncologist [13]. In our case, we had to manage the impaired renal function and also facilitate the staging of the disease by restoring the affected organ to its original position. Reduction of the prolapse facilitated the radiotherapy treatment and minimized the risk of visceral injury due to radiation. Although in most cases reported vaginal hysterectomy with bilateral iliopelvic lymphadenectomy has been performed prior to additional treatment, a more conservative approach might be beneficial in cases of severe comorbidity similar to our patient [14, 15].

The curative principle of cervical cancer including radical surgery with vaginal hysterectomy and adjuvant radiotherapy or radiotherapy for early stages and chemoradiotherapy for locally advanced cancer is acceptable in cases where cancer coexists with prolapsed [11, 12, 16]. Most authors indicate radical vaginal hysterectomy with bilateral iliopelvic lymphadenectomy complemented with external pelvic irradiation and chemotherapy [17, 18]. It is understood that the treatment strategy should be based on the clinical stage of the disease, the presence of metastatic disease, and patient's performance status [13]. According to the clinical stage (IIIB) and the general condition of our patient, palliative chemotherapy and radiotherapy were recommended.

## 4. Conclusion

The concurrence of advanced cervical carcinoma and POP is rare and usually affects elderly women. Staging and treatment plan of cervical cancer is hampered by the anatomic dislocation of the affected organ. Our primary concern in our patient was the restoration of kidney function and the palliative care at a later stage. The best therapeutic approach in such cases is yet to be determined. Needless to say, that a multidisciplinary management is mandatory. Reducing the prolapse in order to allow radiation therapy is a viable option in a patient with stage IIIB cervical cancer who is disqualified from surgery due to the severity of the disease.

## Data Availability

There is no underlying data.
